# Open-source and low-cost miniature microscope for on-site fluorescence detection

**DOI:** 10.1016/j.ohx.2024.e00545

**Published:** 2024-06-15

**Authors:** Michio Kawai, Haruka Oda, Hisatoshi Mimura, Toshihisa Osaki, Shoji Takeuchi

**Affiliations:** a Department of Mechano-Informatics, Graduate School of Information Science and Technology, The University of Tokyo, Tokyo, Japan; b Institute of Industrial Science (IIS), The University of Tokyo, Tokyo, Japan; c Artificial Cell Membrane Systems Group, Kanagawa Institute of Industrial Science and Technology, Kanagawa, Japan

**Keywords:** Fluorescence microscope, Open-source, Biohybrid sensor, Raspberry Pi

## Abstract

The development of a compact and affordable fluorescence microscope can be a formidable challenge for growing needs in on-site testing and detection of fluorescent labeled biological systems, especially for those who specialize in biology rather than in engineering. In response to such a situation, we present an open-source miniature fluorescence microscope using Raspberry Pi. Our fluorescence microscope, with dimensions of 19.2 × 13.6 × 8.2 cm^3^ (including the display, computer, light-blocking case, and other operational requirements), not only offers cost-effectiveness (costing less than $500) but is also highly customizable to meet specific application needs. The 12.3-megapixel Raspberry Pi HQ Camera captures high-resolution imagery, while the equipped wide-angle lens provides a field of view measuring 21 × 15 mm^2^. The integrated wireless LAN in the Raspberry Pi, along with software-controllable high-powered fluorescence LEDs, holds potential for a wide range of applications. This open-source fluorescence microscope offers biohybrid sensor developers a versatile tool to streamline unfamiliar mechanical design tasks and open new opportunities for on-site fluorescence detections.

## Graphical abstract

**Figure:**
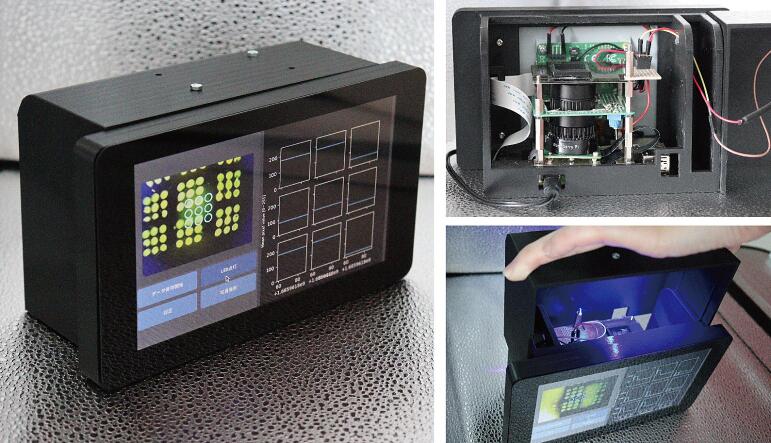

## 1 Specifications table

**Table:** 

Hardware name	**Open-source miniature fluorescence microscope**
Subject area	● *Bioengineering* ● *Engineering and Material science* ● *Educational tools and open-source alternatives to existing infrastructure*
Hardware type	*Imaging tools*
Closest commercial analog	*Fluorescent microscope*
Open source license	Creative Commons Attribution 4.0 International
Cost of hardware	500$
Source file repository	*All supporting software and design files are available at*https://doi.org/10.5281/zenodo.8331548
OSHWA certification UID *(OPTIONAL)*	

## 2 Hardware in context

In the field of fluorescence-based detection and characterization of biological systems [1], [2], [3], [4], [5], [6], [7], [8], [9], ranging from microscopic cells [1], [2] and viruses [3], [4], [5] to small insects [6], there is a growing demand for on-site testing. This involves the identification and analysis of signals emitted by these diverse biological entities through the use of fluorescent molecules. Many practical applications, such as environmental bacteria detection [10], [11] and disease diagnostics through testing [12], [13], require accessible and portable fluorescence microscopes. However, traditional laboratory-grade fluorescence microscopes are often unsuitable for on-site use due to their size and cost limitations. Consequently, there is ongoing research into developing custom-made fluorescence microscopes [14], [15], [16], [17], [18], [19]. On the other hand, many of the custom-made fluorescence microscopes cannot be classified as low-cost or compact because they rely on external computer resources, including laptops and smartphones, and require darkroom setups. Furthermore, their internal architecture and construction often prove unfamiliar to researchers, particularly biologists [20]. This situation presents significant barriers to their utilization in various on-site testing applications.

Here we propose the design of a low-cost (< $500), compact, and open-source fluorescence microscope capable of capturing fluorescent signals from cells. The direct programmability of the Raspberry Pi and the versatility of interchangeable components, including filters, LEDs, and lenses, collectively offer a platform that can be easily customized for specific applications. The amalgamation of affordability, open-source characteristics, and customization potential in this equipment holds the promise of significantly simplifying the development process for point-of-care testing.

## 3 Hardware description

• Enables manufacture of a miniature fluorescent microscope for <$500;
• Provides an easy-to-use front-end interface without the need for peripheral devices;
• Software-controllable LED and camera enable customization as per necessity.

### 3.1 Materials and mechanical design

**Fig. 1:**
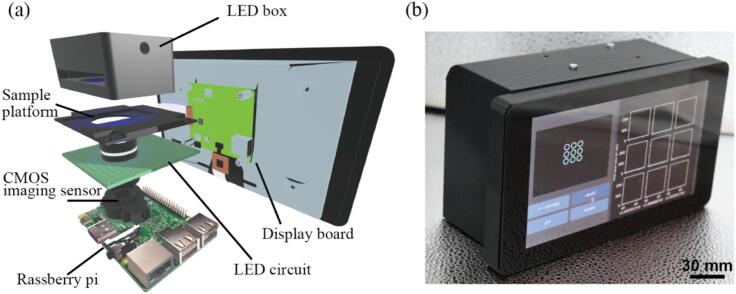
(a) Mechanical design of our proposed device (b) Outlook of our proposed device.

Fig. 1(a) illustrates the mechanical design of the open-source miniature fluorescence microscope that we developed. This device is constructed based on a camera setup utilizing the Raspberry Pi 4 Model B (Raspberry Pi) and the Raspberry Pi HQ Camera (HQ camera). The CMOS sensor (IMX477, Sony) used in the HQ camera combines high sensitivity (250 LSB at an exposure time of 1/120 s), high resolution (an active pixel count of 4056(H) × 3040(V), approximately 12.33 M pixels), and affordability, making it suitable for this application. The lens employed is the 6 mm Wide Angle Lens for Raspberry Pi HQ Camera CS (wide-angle lens), which offers a wide field of view and easy focus adjustment. Assuming a standard focal length of 160 mm following the conventional magnification definition in standard microscope hardware, the magnification of this lens corresponds to approximately 26.7×. Naturally, this lens can be replaced with any CS-mount lens of your choice to match specific applications.

At the top of the lens, there is a platform where the sample for observation is positioned. From above this platform, illumination light is projected onto the sample from the LED box. The LED box is equipped with a slot for inserting filters for adjusting the illumination wavelength. This allows for the installation of custom filters according to the specimen being observed. Additionally, a filter for light reception is positioned between the image sensor and the lens.

Fig. 1(b) shows the outlook of the device. This device is enclosed within an enclosure crafted through 3D printing, which effectively shields it from ambient light. Operational control is facilitated through an integrated touch display affixed to the front panel. This architectural configuration endows the microscope with the unique capability of autonomous usage, obviating the necessity of a darkroom setup or external computing resources.

### 3.2 Electronics

**Fig. 2:**
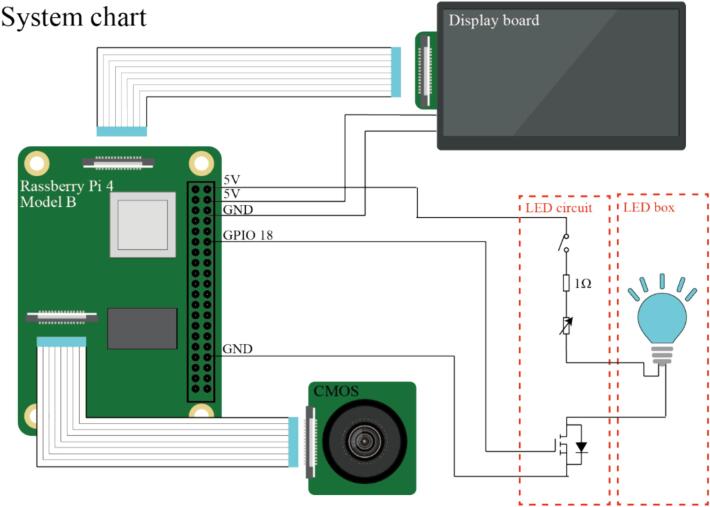
Connection diagram of the system.

Fig. 2 illustrates the connection diagram of this device. The circuitry of this device can be broadly categorized into the Raspberry Pi main unit, display board, HQ camera board, and LED circuit. Power is supplied to the Raspberry Pi 4 through a 12 V DC source, and subsequently distributed to the display and LED circuits via the 5 V output pin. The display circuit is linked to the Raspberry Pi through the Display Serial Interface (DSI). Similarly, the camera board is connected to the Raspberry Pi via the Mobile Industry Processor Interface (MIPI).

The LED circuit comprises a fixed resistor for current limiting, a variable resistor for brightness adjustment, a hardware switch, and a software-controlled switch implemented using a Field-Effect Transistor (FET). The variable resistor enables the adjustment of LED brightness by rotating its knob. The software-controlled switching of LED illumination and deactivation is facilitated by the operation of a general-purpose Input/Output (GPIO) pin connected to the gate of the FET.

### 3.3 Software description

We used Raspberry Pi OS (Legacy), a port of Debian Buster with security and desktop environment, as the operating system for the Raspberry Pi.

One significant advantage of this device is that it can be easily customized and modified to suit specific needs or requirements. As an illustration, we are sharing an open-source program to measure and export the temporal changes of average pixel values within nine regions on the screen.

## 4 Design files summary

**Table:** 

**Design file name**	**File type**	**Open source license**	**Location of the file**
Schematic	Figure	Creative Commons Attribution 4.0 International	https://doi.org/10.5281/zenodo.8331548
3D Design files	CAD files	Creative Commons Attribution 4.0 International	https://doi.org/10.5281/zenodo.8331548
Document of LED	PDF file		https://docs.rs-online.com/0c7b/0900766b812dc602.pdf
Document of filters	PDF files		https://www.asahi-spectra.co.jp/r_filter/filter_detail.asp?key_1=LV0530
Document of Camera	Web page		Raspberry Pi Documentation - Camera
Document of Lens	Web page		https://datasheets.raspberrypi.com/hq-camera/hq-camera-product-brief.pdf
Python source code	Software	Creative Commons Attribution 4.0 International	https://doi.org/10.5281/zenodo.8331548

## 5 Bill of materials summary

**Table:** 

**Designator**	**Component**	**Number**	**Cost per unit −currency**	**Total cost −currency**	**Source of materials**	**Material type**
CPU	Raspberry Pi 4 Model B/ 8 GB	1	75$	75$	PiShop.US	Electronics
Camera	Raspberry Pi HQ Camera	1	50$	50$	PiShop.US	Electronics
Display	Official Raspberry Pi 7″ Touch Screen Display with 10 Finger Capacitive Touch	1	60$	60$	PiShop.US	Electronics
Lens	6 mm Wide Angle Lens for Raspberry Pi HQ Camera CS	1	31.25$	31.25$	PiShop.US	Electronics
SD card	MicroSD Card − 32 GB − Class 10 − BLANK	1	9.95$	9.95$	PiShop.US	Electronics
Filter in lens	LV0530	1	100$	100$	Asahi Spectra Co., Ltd.	Optics
Filter in LED box	SV0490	1	100$	100$	Asahi Spectra Co., Ltd.	Optics
LED board	AE-RasPi-Universal	1	Less than 2$	Less than 2$	AKIZUKI DENSHI TSUSHO CO., LTD.	Electronics
Others	Other Electronic components	1	Less than 5$	Less than 5$	AKIZUKI DENSHI TSUSHO CO., LTD.	Electronics

The RAM capacity of the Raspberry Pi is contingent upon the computational demands of the employed software. For tasks involving observation and simple calculations, even the minimum capacity of 1 GB (priced at $35) is adequate to ensure smooth operation. The listed prices are based on individual pricing from major global distributors. Utilizing different local distributors or exploring bundle pricing options can potentially lead to even more cost-effective assembly. “Other Electronic Components” refers to the variable resistor (∼500 Ω) utilized in the LED circuit, current-limiting resistor, Field-Effect Transistor (FET), and jumper wires. These components do not have particularly stringent specifications, and as long as the acceptable current limits are taken into account, the selection of components can be made based on availability and convenience.

## 6 Build instructions

This device is structured with five layers: the Raspberry Pi, HQ camera and lens, LED circuit board, sample platform, and LED Box, all stacked using male and female screws and enclosed within the enclosure. The lens attached to the camera layer penetrates through the LED circuit layer.

**Fig. 3:**
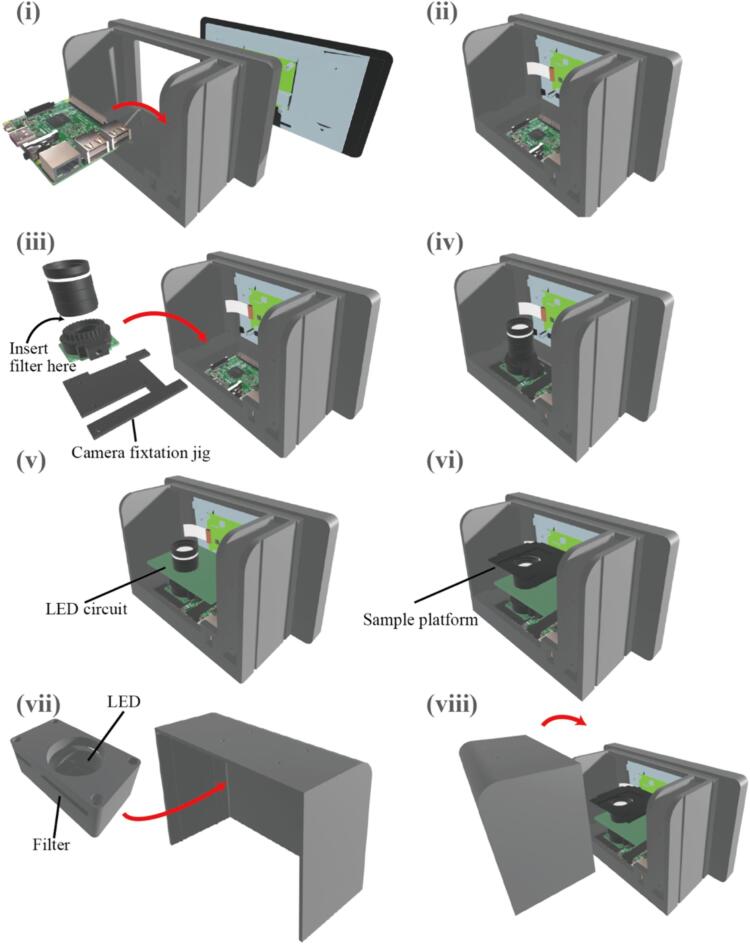
Assembly steps of our proposed device.

1. Securing the Raspberry Pi and Display (Fig. 3(i–ii)):

Begin by attaching the Raspberry Pi inside the enclosure at the designated location. Use appropriate screws to secure the Raspberry Pi to the enclosure's bottom. Then, attach the display onto the front face of the enclosure. Use suitable screws to fasten the display securely to the enclosure.

Utilize a flexible cable to establish a connection between the display and the Raspberry Pi. Insert the cable connector into the corresponding port on the display board.

Ensure the supply of power between the Raspberry Pi and the display board by connecting the 5 V power and ground (GND) wires. This step guarantees that display board can receive the necessary power to operate effectively.

2. Positioning the HQ Camera using a Mounting Fixture (Fig. 3(iii–iv)):

Following the previous steps, after assembling the Raspberry Pi and the display, proceed to attach the HQ Camera using a camera mounting fixture with screw holes. The HQ Camera should be positioned on the top surface of the Raspberry Pi. Use male and female screws to secure the HQ Camera in place by passing them through the screw holes in the mounting fixture and the camera board.

Use a flexible cable to establish a connection between the HQ Camera module and the Raspberry Pi. This flexible cable serves to transmit data and signals between the camera board and the Raspberry Pi. Make sure to properly connect the cable's connectors to their corresponding ports on both the camera board and the Raspberry Pi.

Inside the HQ Camera module, arrange the light-receiving filter in a way that it completely covers the imaging sensor section. This filter helps enhance the image quality and manage light conditions. Additionally, mount a wide-angle lens onto the HQ Camera module. This lens should be attached on top of the filter, ensuring optimal focus and image capture.

3. Positioning the LED circuit layer (Fig. 3(v)):

Assemble the LED circuit as illustrated in Fig. 2. Use a universal PCB (printed circuit board) to integrate components such as FET (Field-Effect Transistor), HW switch, fixed resistors for current limitation, and potentiometers for adjustable resistance. To minimize noise, consider using a black universal PCB or applying a coating with black ink.

Position the LED circuit layer using male and female screws. At this point, make sure to drill a hole in universal PCB that allows the lens to pass through.

4. Positioning the sample platform (Fig. 3(vi)):

Position the sample platform using male and female screws to ensure an appropriate distance between the lens and the sample. The sample platform in this paper is designed to accommodate a cover glass of 24 mm x 60 mm, but it is recommended to modify the platform's design to suit the object of interest you wish to observe.

Fine focus adjustments can be made by turning the knob on the wide-angle lens for precise focal length adjustments.

5. Creating and securing the LED Box (Fig. 3(vii)):

Attach LEDs onto the LED box and insert the filter into the slit to adjust the illumination wavelength. Wiring of the LEDs can be easily managed by routing the wires through holes located on the side of the enclosure. The assembled LED Box is secured on the cover using screws.

6. Fitting the cover onto the enclosure (Fig. 3(viii)):

The device can be assembled by placing the cover onto the enclosure following the guide.

## 7 Operation instructions

1. Plug in the power and start the system.
2. Download and execute “Application.py” on the Raspberry Pi. If there are missing libraries, connect the Raspberry Pi to the internet and download them. The required libraries are as follows: NumPy, matplotlib, opencv-python, custom TK-inter.
3. A screen as Fig. 1B will appear, and the measurement will commence

Press the LED button to activate the LED. If you wish to adjust the brightness, you can do so by turning the knob of the variable resistor on the LED circuit layer.

## 8 Validation and characterization

### 8.1 Evaluation of the distortion

**Fig. 4:**
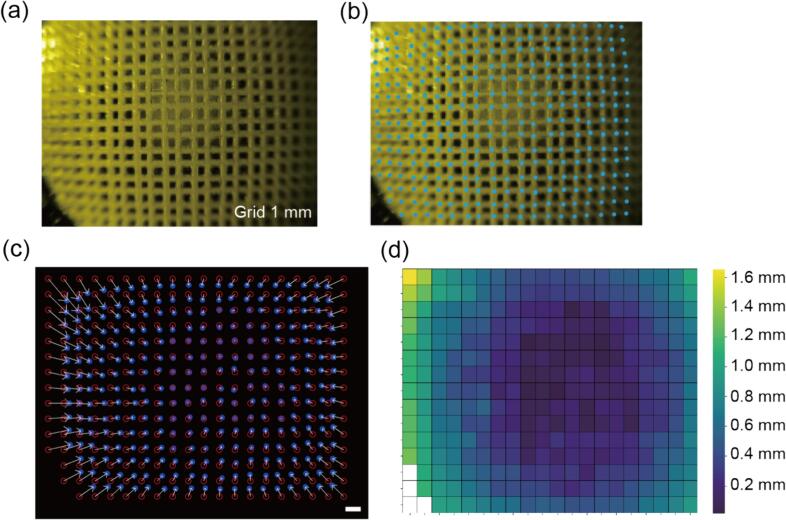
Evaluation of the radial distortion of the wide-angle lens (a) The fluorescent image of the grid line taken by our proposed device. (b) Grid intersections are shown in blue dots. (c) Arrows indicating the displacement of each grid after distortion. (d) Heatmap representing the amount of displacement of each grid after distortion. Scale bar, 1 mm. (For interpretation of the references to colour in this figure legend, the reader is referred to the web version of this article.)

The wide-angle lens used in this microscope allows for a broad range of fluorescence signals, while also causing radial distortion. This radial distortion was quantitatively assessed using a 1 mm-grid-patterned acrylate chip. A chip was fabricated by grinding a 1 mm grid using a 0.2 mm drill and subsequently, the fluorescent specimen was poured into the grid. As illustrated in Fig. 4(a), the field of view measured 21 mm x 15 mm, with the defocusing observed at the outer periphery. The cross points of the grid were detected using MATLAB software, and the remaining points that could be identified were manually supplemented (Fig. 4(b)). Subsequently, the grid's original position was calculated by centering it on the grid point nearest to the center of the image. Arrows representing the displacement between the original position and the position in the distorted image were then depicted in Fig. 4(c). The displacement length for each grid was quantified as a heatmap in Fig. 4(d). Due to distortion, the image edges showed a maximum displacement of approximately 1.6 mm. While distortion does not pose an issue when evaluating only the fluorescence response or intensity, it is important to consider this distortion when assessing the shape.

### 8.2 Comparison of the performance between our proposed device and the laboratory-grade fluorescence microscope

To evaluate the device’s capability for capturing the fluorescence signals, fluorescence sensitivity of the system was validated. The investigation of the fluorescent range detectable by the device involved the creation of a spatial array by puncturing a 1.5 mm hole in a 1 mm thick PDMS sheet. An initial 0.125 vol% solution of fluorescent dye (Fluoresbrite® YG Microspheres 0.50 µm, Polyscience) was prepared and subsequently diluted. This resulted in 25 distinct concentration solutions: concentrations ranging from 10 % to 100 % in 10 % increments, from 1 % to 9 % in 1 % increments, and from 0.4 % to 0.9 % in 0.1 % increments of the initial concentration. The 25 distinct concentration solutions were individually applied to the punctured spots, forming an array. Subsequent observation of this array was conducted using a fluorescent microscope (IX73, Olympus) with 1.25 × lens (NA0.04, PlanApoN), excitation (U-LGPS, Olympus), and a mirror cube for filtering (U-FBW, Olympus) in conjunction with our proposed device, facilitating a comprehensive assessment of the device's fluorescence detection capabilities.

**Fig. 5:**
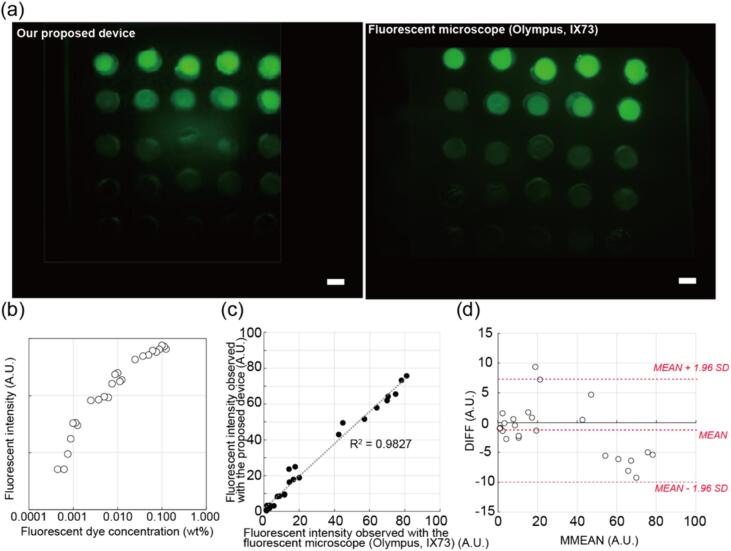
Comparison of the performance between our proposed device and the fluorescence microscope (a)The fluorescence image of the array of fluorescent dye solutions with different concentrations. The image on the left was taken by our proposed device, and the image on the right was taken by the fluorescent microscope. (b)The relationship between the fluorescent intensity calculated from the taken image of our proposed device and the concentration of the fluorescent dye. (c)The comparison of fluorescent intensity measured by our proposed device and the fluorescent microscope. (d)Brand-Altman plot comparing the performance of our proposed device and the fluorescent microscope. Scale bars, 1 mm.

The fluorescence images obtained from our proposed device and the fluorescent microscope and are shown in Fig. 5(a). The acquired images were then subjected to analysis using ImageJ (Rasband, W.S., ImageJ, U. S. National Institutes of Health, Bethesda, Maryland, USA, https://imagej.nih.gov/ij/, 1997–2018.) software, which allowed us to determine the fluorescence intensity values for each concentration level (Fig.5(b)). To evaluate the precision and reliability of our device, we calculated the correlation between the fluorescent intensity values calculated from the fluorescent microscope and the corresponding measured fluorescence intensities from this device (Fig. 5(c)). This analysis yielded an R^2^ value of 0.98, indicating a strong linear relationship between the two variables, affirming its accuracy and reliability. We used a Bland-Altman plot to further compare the performance of our proposed device with that of the fluorescent microscope (Fig.5(d)). The 95 % confidence interval for the mean difference between the two measurement methods is −3.15 to 0.45, indicating that the mean difference is close to zero. Additionally, over 95 % of the data points (24/25) fall within the 95 % limits of agreement (mean ± 1.96 SD). Thus, it can be concluded that the two measurement methods have equivalent performance under the given conditions. These results highlight the device's capacity for accurate and consistent fluorescence measurements across a broad range of concentrations, equivalent to a fluorescence microscope with a 1.25 × lens. Although the performance of our proposed device may not match that of a fluorescence microscope with higher magnification lenses, it offers sufficient performance when considering its affordability and portability.

### 8.3 Demonstration of the biohybrid sensing

To demonstrate the potential of this sensor as a bio-hybrid sensor, we conducted odorant detection tests using sensor cells responsive to a specific odorant. Sensor cells responsive to an odorant, acetophenone, were established from cultured insect cells (ExpiSf9, Thermo Fisher Scientific) and kindly provided by SUMITOMO CHEMICAL (Japan). The sensor cells constitutively express components of the insect olfactory receptor system, including a chimeric co-receptor (DmAmOrco, derived from *Drosophila melanogaster* and *Apis mellifera*) and an olfactory receptor (AaOR15, from *Aedes aegypti*) together with the calcium indicator green fluorescence protein (GCaMP3).

**Fig. 6:**
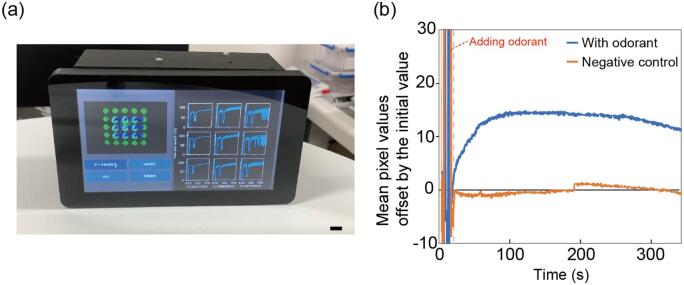
Demonstration of the device as a biohybrid sensor using sensor cells (a) Image of the device during measurement (b) Fluorescence intensity change of sensor cells in response to odorant. Scale bar, 10 mm.

The sensor cells were encapsulated in a collagen hydrogel (Nitta Gelatin, Japan) and spotted onto an acrylic substrate at a volume of 1 µl per spot. The cells were maintained in a Hanks' Balanced Salt Solution (HBSS, Thermo Fisher Scientific) buffered with 20 mM 4-(2-hydroxyethyl)-1-piperazineethanesulfonic acid (HEPES) at pH 7.2, containing 0.1 % bovine serum albumin (BSA). The odorant, acetophenone dissolved in dimethyl sulfoxide (DMSO), was diluted in the HBSS/HEPES solution and applied to the cells on the substrate at a final concentration of 100 µM, with 1 % DMSO. As a negative control, HBSS/HEPES with 1 % DMSO was used. Using the developed imager, a series of fluorescence intensities were collected sequentially at the designated spots every 300 ms. The odorant solution or the negative control solution was applied by pipetting 30 s after starting the imaging. The intensities in three spots were averaged and plotted as shown in Fig. 6. In the sample where the odorant was applied, the mean pixel value of the sensor cells that detected the odorant increased by approximately 15, followed by a gradual decrease. This result demonstrates the potential of this microscope device for the biohybrid sensing.

### 8.4 Demonstration of the customizability

**Fig. 7:**
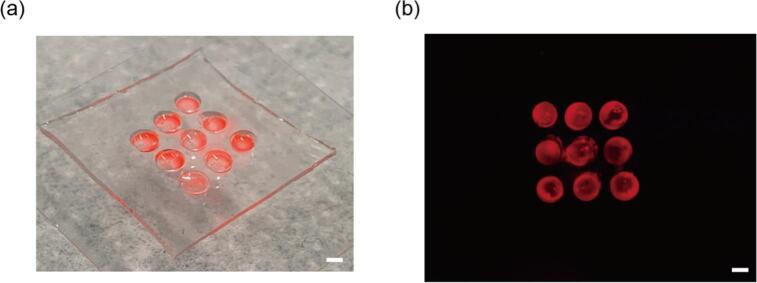
Observation of red fluorescence by exchanging the filter (a) Image of the device used for observation. (b) Observation of red fluorescent beads through the replaced filter. Scale bars, 1 mm. (For interpretation of the references to colour in this figure legend, the reader is referred to the web version of this article.)

We demonstrated the customizability of the device by changing the filter for the detection of different fluorescent specimens. We replaced the filter in lens with a red long-pass filter (XF3074, OMEGA® Optical) and observed the red fluorescent beads. For the observation, we prepared a spatial array by puncturing holes in a thick PDMS sheet. A solution of 0.005 g/ml red fluorescent dye (FMR − Red Fluorescent Polymer Microspheres 1.3 g/cc, 1-5um, Cospheric) was prepared and poured into the punctured spots, forming an array (Fig. 7a). Subsequent observation of this array was conducted by our proposed device (Fig. 7b). By changing the filter, we demonstrated that it is possible to observe different fluorescent colors, showcasing the customizability of our proposed device.

## 9 Ethics statements

No human and animal subjects are used in this research.

## CRediT authorship contribution statement

**Michio Kawai:** Writing – original draft, Methodology, Investigation. **Haruka Oda:** Writing – review & editing, Writing – original draft, Investigation. **Hisatoshi Mimura:** Investigation, Writing – original draft, Writing – review & editing. **Toshihisa Osaki:** Funding acquisition, Supervision, Writing – review & editing. **Shoji Takeuchi:** Writing – review & editing, Project administration, Funding acquisition, Conceptualization.

## Declaration of competing interest

The authors declare that they have no known competing financial interests or personal relationships that could have appeared to influence the work reported in this paper.
